# Adding customized electron energy beams to TrueBeam linear accelerators

**DOI:** 10.1002/acm2.13633

**Published:** 2022-05-09

**Authors:** Song Gao, Manickam Muruganandham, Weiliang Du, Jared Ohrt, Rajat J. Kudchadker, Peter A. Balter

**Affiliations:** ^1^ Department of Radiation Physics The University of Texas MD Anderson Cancer Center Houston Texas USA

**Keywords:** acceptance and commissioning, ionization chamber array, MPPG 5.a tests, new energy electron beams

## Abstract

**Purpose:**

To better meet clinical needs and facilitate optimal treatment planning, we added two new electron energy beams (7 and 11 MeV) to two Varian TrueBeam linacs.

**Methods:**

We worked with the vendor to create two additional customized electron energies without hardware modifications. For each beam, we set the bending magnet current and then optimized other beam‐specific parameters to achieve depths of 50% ionization (*I*_50_) of 2.9 cm for 7 MeV and 4.2 cm for the 11 MeV beam with the 15 × 15 cm^2^ cone at 100 cm source‐to‐surface distance (SSD) by using an ionization chamber profiler (ICP) with a double‐wedge (DW) phantom. Beams were steered and balanced to optimize symmetry with the ICP. After all parameters were set, full commissioning was done including measuring beam profiles, percent depth doses (PDDs), output factors (OFs) at standard, and extended SSDs. Measured data were compared between the two linacs and against the values calculated by our RayStation treatment planning system (TPS) following Medical Physics Practice Guideline 5.a (MPPG 5.a) guidelines.

**Results:**

The *I*_50_ values initially determined with the ICP/DW agreed with those from a PDD‐scanned in‐water phantom within 0.2 mm for the 7 and 11 MeV on both linacs. Comparison of the beam characteristics from the two linacs indicated that flatness and symmetry agreed within 0.4%, and point‐by‐point differences in PDD were within 0.01% ± 0.3% for the 7 MeV and 0.01% ± 0.3% for the 11 MeV. The OF ratios between the two linacs were 1.000 ± 0.007 for the 7 MeV and 1.004 ± 0.007 for the 11 MeV. Agreement between TPS‐calculated outputs and measurements were −0.1% ± 1.0% for the 7 MeV and 0.2% ± 0.8% for the 11 MeV. All other parameters met the MPPG 5.a's 3%/3‐mm criteria.

**Conclusion:**

We were able to add two new beam energies with no hardware modifications. Tuning of the new beams was facilitated by the ICP/DW system allowing us to have the procedures done in a few hours and achieve highly consistent results across two linacs.

PACS numbers: 87.55.Qr, 87.56.Fc

## 1 INTRODUCTION

The number of beam energies on the Varian Clinac platform is limited by machine design, with control slots for beam tuning in which each slot is tied to settings such as bending magnet current and carriage position.^1^ Because these data are maintained via software in TrueBeam platform, we were able to work with the vendor to add two new beam energies to the existing energies, with the only limitation being that the beams need to share scattering foils with the existing energies.

Varian TrueBeam linear accelerators (linacs) are offered with 6‐, 9‐, 12‐, 15‐, 16‐, 18‐, 20‐, and 22‐MeV electron beam energies. The “standard” electron beams historically utilized in our clinic are 6, 9, 12, 16, and 20 MeV. The spacing of the therapeutic depths, *R*_80_ (80% maximum dose) and *R*_90_ (90% maximum dose) obtained from percent depth dose (PDD) measurements (with a 10 × 10 cm^2^ cone at a source‐to‐surface distance [SSD] of 100 cm) are about 1.0 cm between the 6‐ and 9‐MeV beams and between 9‐ and 12‐MeV beams. But our clinic prefers to use a spacing of 0.5 cm with intermediate energy beams to provide optimal planning flexibility and dose conformity, especially to treat internal mammary chain (IMC) lymph nodes in locally advanced breast cancer patients. Our 3D conformal treatment plans use a medial IMC electron field matched with tangential photon beams. The *R*_90_ of the electron fields just covers the IMC nodes, whereas the sharp dose falloff beyond *R*_90_ significantly minimizes the dose to underlying heart and lung structures. Therefore, available electron energies with 0.5‐cm increments of *R*_90_ would facilitate optimal treatment planning in terms of sparing ipsilateral lung and heart and having a uniform dose coverage of target.^1^

We worked with the vendor to implement two additional electron beam energies, 7 and 11 MeV, to fill the gaps between our existing energies.

The two beam energies were commissioned on two linacs as follows. Treatment planning models of the two beams were generated and commissioned in the RayStation treatment planning system (TPS) and those TPS beam models were validated based on AAPM Medical Physics Practice Guideline 5.a (MPPG 5.a).^2^ These two beams were also commissioned and validated for the RadCalc monitor unit calculation software (Lifeline Software Inc, Tyler, TX), which is used to verify the secondary dose calculation.

The purpose of this study was to implement two intermediate‐energy 7‐ and 11‐MeV electron beams on both TrueBeam linacs to facilitate optimal treatment planning dosimetry. Comprehensive commissioning measurements and validation tests indicated that the new electron beams met all dosimetry parameter criteria specified by MPPG 5.a.

## 2 MATERIALS AND METHODS

The new 7‐MeV beam shared the scattering foil of the 6‐MeV beam, and the new 11‐MeV beam shared the scattering foil of the 9‐MeV beam. Implementation procedures are described in the following section.

### 2.1 Addition of new energy beams to TrueBeam linacs

Each linac was steered with specific waveguide radio‐frequency power and beam parameters to maximize the electron beam output (dose rate). The bending magnet current was adjusted to give depths of 50% of the maximum beam ionization (*I*_50_) for a 15 × 15‐cm^2^ cone of 2.9 cm for the 7‐MeV beam and 4.2 cm for the 11‐MeV beam. The energy was set by using an ionization chamber profiler (ICP) and double‐wedge (DW) phantom that were calibrated with standard electron beams.^3^ The beams were steered and balanced to optimize symmetry with the ICP.^4^ These procedures were performed on both linacs to achieve matched electron beams on two linacs. After these electron energy beams were successfully implemented, we performed acceptance tests such as electron applicators preset sizes versus beam energies specified by manufacturer and beam stability (output and dose rate) over full gantry rotation. Beam energy (depth ionization) and profile verification were done with a 3D water phantom at 100‐cm SSD; the depth Ionizations were scanned with a 15 × 15‐cm^2^ cone size specified by manufacturer. The *I*_50_ values were compared with those initially set using the ICP/DW system. Beam profiles were measured with both 10 × 10‐ and 25 × 25‐cm^2^ cones at a depth of *I*_85%_/2; the flatness and symmetry were calculated from the profiles for the beam centerline within the central 80% of the in‐ and cross‐plane axes such that the symmetry does not exceed 2.0% for all beams and measurement geometries.

### 2.2 Commissioning the new beams

Beam profiles, depth‐ionizing curves, and output factors (OFs) were measured by using water phantoms for various cones/cutout geometries as follows.

#### 2.2.1 Percent depth dose

The depth‐ionizing curves were scanned in water with 100‐cm SSD for cone/cutout sizes from 2 × 2 to 25 × 25 cm^2^, and the PDDs were then obtained according to the AAPM TG‐25 protocol. We compared the depths of the maximum dose (*d*_max_) and that at 90%, 80%, and 50% of the maximum dose for both linacs. The average values of the PDDs of the two linacs were used for RayStation TPS modeling and RadCal secondary monitor unit check software commissioning. The point‐by‐point differences between average and measured PDDs were also compared. The PDDs (10 × 10‐cm^2^ cone) of the new 7‐ and 11‐MeV electron beams were compared with the existing adjacent 6‐ and 12‐MeV beams. The open field (without applicator) PDD at 100‐cm SDD with jaws retracted for each beam was measured for RayStation TPS modeling.

#### 2.2.2 Beam profile

We measured the beam profile in water with an SSD of 100 cm by using a 3D water phantom at different depths of *d*_max_, *R*_90%_ and *R*_50%_ for all standard 6 × 6‐, 10 × 10‐, 15 × 15‐, 20 × 20‐, and 25 × 25‐cm^2^ cones along the in‐ and cross‐plane directions. Flatness was defined as the maximum variation in integrated dose between the minimum and maximum points within the central 80% of the in‐ and cross‐plane major axes profiles. Symmetry was defined as the maximum variation in integrated dose between any two corresponding points equidistant from the beam centerline within the central 80% of the in‐ and cross‐plane profiles. The beam profiles (at a depth of *I*_85_/2) of the new 7‐ and 11‐MeV electron beams were compared with the existing adjacent 6‐ and 12‐MeV beams. We also measured the beam profile in water with an SSD of 100 cm at deeper depths (Bremsstrahlung region) of 5.5 cm for 7‐MeV and 7.2 cm for 11‐MeV beams, all with standard cones, for the purpose of RayStation (version 10A) TPS modeling with Monte Carlo algorithm. We also measured the beam profiles in air without an applicator for different field sizes at two different SSDs as recommended in the RayStation TPS modeling documentation; the details are given in Section 4.

#### 2.2.3 Output factors

The OFs were measured at *d*_max_ for each cutout in a 1D water phantom for cone/cutout sizes of 2 × 2, 3 × 3, 4 × 4, 6 × 6, 8 × 8, 10 × 10, 15 × 15, 20 × 20, and 25 × 25 cm^2^ and for SSDs of 100, 105, 110, 115, and 120 cm. The OF was the ratio of the output with given cone/cutout and SSD to the output with the standard 10 × 10‐cm^2^ cone at an SSD of 100 cm. The average of the OFs for all cone/cutout combinations of the two linacs were used for TPS modeling and RadCal commissioning. The ratio differences between measured OFs from the two linacs and the averaged OFs were compared. The OFs for different cone/cutout combinations were also compared.

### 2.3 TPS modeling and validation

Models of the new beams were created in the RayStation TPS. The measured data were compared against the TPS‐calculated values according to MPPG 5.a. recommendations.^2^ The following basic beam validation tests included (a) the PDD comparison for field sizes of 2 × 2, 4 × 4, 5 × 5, 6 × 6, 10 × 10, 15 × 15, 20 × 20, and 25 × 25 cm^2^ and profile comparison for standard cones at standard SSD, and (b) the OF comparison of various cone/cutout combinations at a reference depth (*d*_max_ depth of the field) for standard and extended SSDs. In addition, testing for surface irregularities obliquity validation was done with the 10 × 10‐cm^2^ cone, and the gantry was angled 20° from perpendicular to a water phantom at SSD of 100 cm. For the beam oblique incidence tests, the beam profile and point dose were compared between measurements and TPS calculations in a low‐ and a high‐gradient region. The percentage difference was used to indicate the differences between TPS‐calculated and measured data in the flat region of profiles and the region from surface to *d*_max_ of PDD data as well as in the outside field profile region and the Bremsstrahlung region of the PDD. The distance‐to‐agreement (DTA) was used to indicate the difference in the penumbra region of profiles and the deep falloff region of the PDD. For normal incidence tests, the MPPG 5.a recommends that 3% agreement in the high‐dose region/low‐dose gradient and 3.0‐mm DTA for PDDs along the central axis. For oblique incidence tests, the MPPG 5.a recommends a tolerance of 5% for high‐dose/low‐gradient regions and a DTA of 3.0 mm for high‐gradient regions.

### 2.4 Commissioning of RadCalc monitor unit calculation software

We used the RadCalc monitor unit calculation software for secondary dose calculation verification. The OFs of the five standard open cones measured at 100‐cm SSD and the OFs of each cone/cutout combination at SSDs from 100 to 120 cm are required for these tests. The effective SSD values were calculated for each cone by using measured OFs in water at different SSDs (from 100 to 120 cm) by using the method described by Khan et al.^5^ The effective SSD is used within RadCalc to remove the inverse square effect from the cutout‐specific OFs, which enables more accurate linear interpolation. The PDD curves for all cone/cutout combinations of the two beams were as follows: 7 MeV: 2 × 2, 3 × 3, 4 × 4, 5 × 5, 6 × 6, 10 × 10, and 25 × 25 cm^2^; 11 MeV: 2 × 2, 3 × 3, 4 × 4, 5 × 5, 6 × 6, 8 × 8, 10 × 10, and 25 × 25 cm^2^.

To verify the PDD data, we compared the dose per MU (cGy/MU) calculated with the RadCalc software with that calculated from the measured OFs for various cone/cutout combinations at 100‐cm SSD and depths from 1.0 cm to the practical range of the beams. To verify the MU calculations, we compared the RadCalc‐calculated MUs for various cone/cutout combinations at *d*_ma×_ for SSDs of 100, 105, 110, 115, and 120 cm with those calculated from the measured data.

## 3 RESULTS

### 3.1 Acceptance tests of new beams

The electron applicator preset sizes versus beam energies and beam stability for full gantry rotation met Varian's specifications. The *I*_50_ values from the depth Ionizations curves, scanned in a 3D water phantom with a 15 × 15‐cm^2^ cone, agreed with the *I*_50_ values initially set with the ICP/DW system within 0.2 mm. The depths of ionization *I*_90_, *I*_80_, and *I*_50_ agreed with Varian's specifications within ±0.4 mm (tolerance: ±0.7 mm) across two beams on two linacs (L1 and L2) (Table 1).

**TABLE 1:** The depth (cm) of 50% ionization (*I*_50_) measured in different methods: initial setup with ICP/DW; then verified using 3D water scans (water)

		*I*_50_ (Spec: 7e: 2.92, 11e: 4.23)				*I*_80_ (Spec: 7e: 2.44, 11e: 3.58)		*I*_90_ (Spec: 7e: 2.20, 11e: 3.25)	
Energy (MeV)	Parameter	L1		L2		L1	L2	L1	L2
	**Method**	ICP/DW	water	ICP/DW	water	Water		Water	
**7**	**Measured**	2.91	2.91	2.91	2.91	2.42	2.40	2.19	2.16
	**Difference**	−0.01	−0.01	−0.01	−0.01	−0.02	−0.04	−0.01	−0.04
**11**	**Measured**	4.21	4.23	4.26	4.25	3.58	3.58	3.25	3.25
	**Difference**	−0.02	0.00	0.03	0.02	0.00	0.00	0.00	0.00

*I*_80_ and *I*_90_ are from 3D water scans. Measurement condition: 15 × 15‐cm^2^ cone at 100‐cm SSD. The difference between measured values and Varian specifications (Spec, in the parentheses) are within ±0.04 cm.

Abbreviations: DW, double‐wedge; ICP, ionization chamber profiler; SSD, source‐to‐surface distance.

Beam profiles were measured with 10 × 10‐ and 25 × 25‐cm^2^ cones at a depth of *I*_85%_/2; the flatness and symmetry (Table 2) were within Varian's specifications (flatness < 4.5%, symmetry < 2%).

**TABLE 2:** Flatness (%) and symmetry (%) measured from profiles scanned with 10 × 10‐ and 25 × 25‐cm^2^ cones at 100‐cm SSD with a 3D water scanning system

7‐MeV flatness and symmetry (%) (depth 1.2 cm)								
	Inline				Crossline			
	10 × 10		25 × 25		10 × 10		25 × 25	
Linac	Flat	Symm	Flat	Symm	Flat	Symm	Flat	Symm
**L1**	2.0	0.2	1.0	0.4	2.3	0.7	1.3	0.6
**L2**	2.4	0.5	1.3	0.5	2.5	0.3	1.5	0.8

11‐MeV flatness and symmetry (%) (depth 1.7 cm)								
	Inline				Crossline			
Linac	10 × 10		25 × 25		10 × 10		25 × 25	
	Flat	Symm	Flat	Symm	Flat	Symm	Flat	Symm
**L1**	1.4	0.5	0.9	0.4	1.3	0.2	0.8	0.4
**L2**	1.6	0.3	0.9	0.7	1.5	0.2	0.6	0.5

Abbreviation: SSD, source‐to‐surface distance.

### 3.2 Beam‐commissioning data

#### 3.2.1 Percent depth dose

**FIGURE 1:**
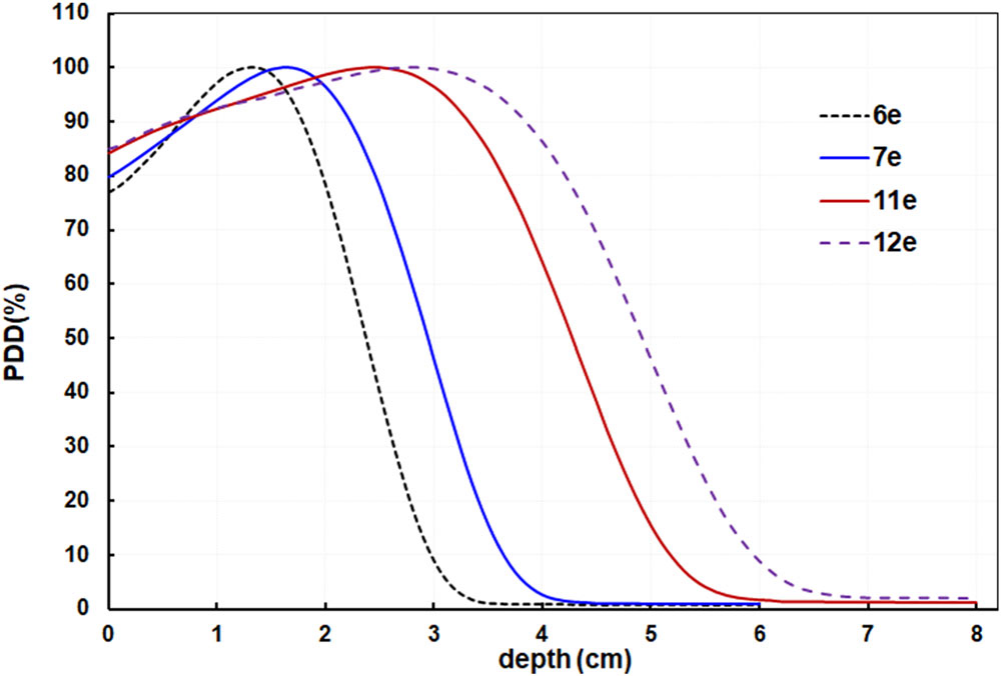
The PDDs of the new 7‐ and 11‐MeV beams compared with their adjacent energies. PDDs, percent depth doses

We compared the PDDs at the depths of *d*_max_, *d*_90%_, *d*_80%_, and *d*_50%_ for both linacs for field sizes ranging from 2 × 2 to 25 × 25 cm^2^ (Table 3) and found that the differences between the two linacs were within 1.0 mm for both the 7‐ and 11‐MeV beams. The point‐by‐point difference–averaged PDDs and L1/L2 were within ±1.0% for both 7‐ and 11‐MeV beams for all measured depths and field sizes. The results indicated that the PDDs of the two beams in the two linacs matched very well. (PDD values and plots vs. field size are described in Supplemental data 1.) Compared to the existing adjacent electron energies, the difference in the depth of the 80% maximum dose was 0.5 cm between the 6‐ and 7‐MeV beams and was 0.5 cm between the 11‐ and 12‐MeV beams (Figure 1).

**TABLE 3:** The depths of maximum dose (*d*_max_), 90% (*R*_90_), 80% (*R*_80_), and 50% (*R*_50_) of the maximum dose for linacs L1 and L2 for field sizes from 2 × 2 to 25 × 25 cm^2^ measured from PDD profiles scanned with a 3D water system at an SSD of 100 cm

**7 MeV**, **L1**										
**Field size (cm^2^)**	**2 × 2**	**3 × 3**	**4 × 4**	**5 × 5**	**6 × 6**	**8 × 8**	**10 × 10**	**15 × 15**	**20 × 20**	**25 × 25**
*d*_max_ (cm)	0.9	1.4	1.6	1.6	1.6	1.6	1.7	1.6	1.6	1.6
*R*_90_ (cm)	1.6	2.1	2.2	2.2	2.2	2.2	2.2	2.2	2.2	2.2
*R*_80_ (cm)	1.9	2.3	2.5	2.5	2.5	2.5	2.5	2.5	2.4	2.4
*R*_50_ (cm)	2.6	2.9	2.9	3.0	3.0	2.9	2.9	2.9	2.9	2.9
**7 MeV, L2**										
*d*_max_ (cm)	0.9	1.4	1.6	1.6	1.6	1.6	1.6	1.6	1.6	1.6
*R*_90_ (cm)	1.6	2.1	2.2	2.2	2.2	2.2	2.2	2.2	2.2	2.2
*R*_80_ (cm)	1.9	2.3	2.5	2.5	2.5	2.5	2.5	2.5	2.5	2.5
*R*_50_ (cm)	2.6	2.9	3.0	3.0	3.0	2.9	2.9	3.0	3.0	3.0
**11 MeV, L1**										
*d*_max_ (cm)	1.1	1.7	2.2	2.4	2.4	2.4	2.4	2.4	2.4	2.5
*R*_90_ (cm)	2.0	2.7	3.1	3.3	3.3	3.3	3.3	3.3	3.3	3.3
*R*_80_ (cm)	2.4	3.1	3.5	3.6	3.6	3.6	3.6	3.6	3.6	3.6
*R*_50_ (cm)	3.4	4.0	4.2	4.3	4.3	4.3	4.3	4.3	4.3	4.3
**11 MeV, L2**										
*d*_max_ (cm)	1.1	1.7	2.2	2.3	2.4	2.4	2.4	2.5	2.5	2.5
*R*_90_ (cm)	2.0	2.7	3.1	3.3	3.3	3.3	3.3	3.3	3.3	3.3
*R*_80_ (cm)	2.4	3.1	3.5	3.6	3.6	3.7	3.6	3.7	3.7	3.7
*R*_50_ (cm)	3.4	4.0	4.2	4.3	4.3	4.3	4.3	4.3	4.3	4.3

Abbreviations: PDD, percent depth dose; SSD, source‐to‐surface distance.

#### 3.2.2 Beam profile

**FIGURE 2:**
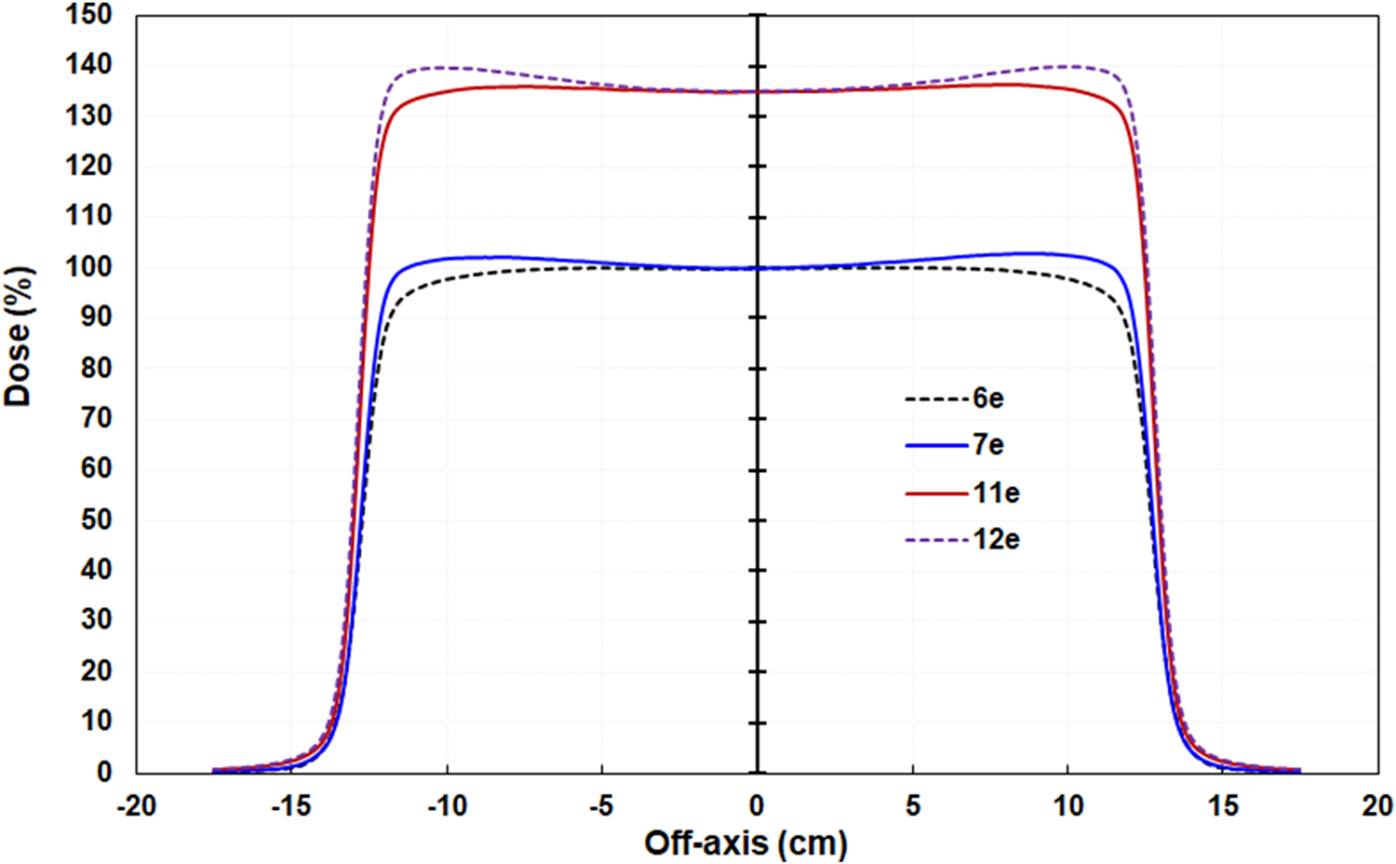
The new 7‐ and 11‐MeV beam profiles compared with their adjacent 6‐ and 12‐MeV beams. Field size: 25 × 25 cm^2^, depths: 1.2 cm for 7 MeV, 1.7 cm for 11‐MeV beams

Beam flatness and symmetry were calculated from in‐ and cross‐plane profiles at three different depths, namely, *d*_max_, *R*_90_ and *R*_50_. Flatness depends strongly on the field size and the depth of profiles, and the beams became less flat at field sizes of ≤10 × 10 cm^2^. The symmetry of the measured profiles taken at depths above the depth of *R*_50_ were 0.6% ± 0.4% (max: 1.5%, min: 0.1%) for the 7‐MeV beam and 0.6% ± 0.3% (max: 1.4%, min: 0.1%) for the 11‐MeV beam. Details of the flatness and symmetry of these two beams for all standard cones at different depths for the two linacs are given in Supplemental data 2. The beam profiles of the new 7‐ and 11‐MeV beams were flatter than their adjacent energy beams (Figure 2).

#### 3.2.3 Output factors

**FIGURE 3:**
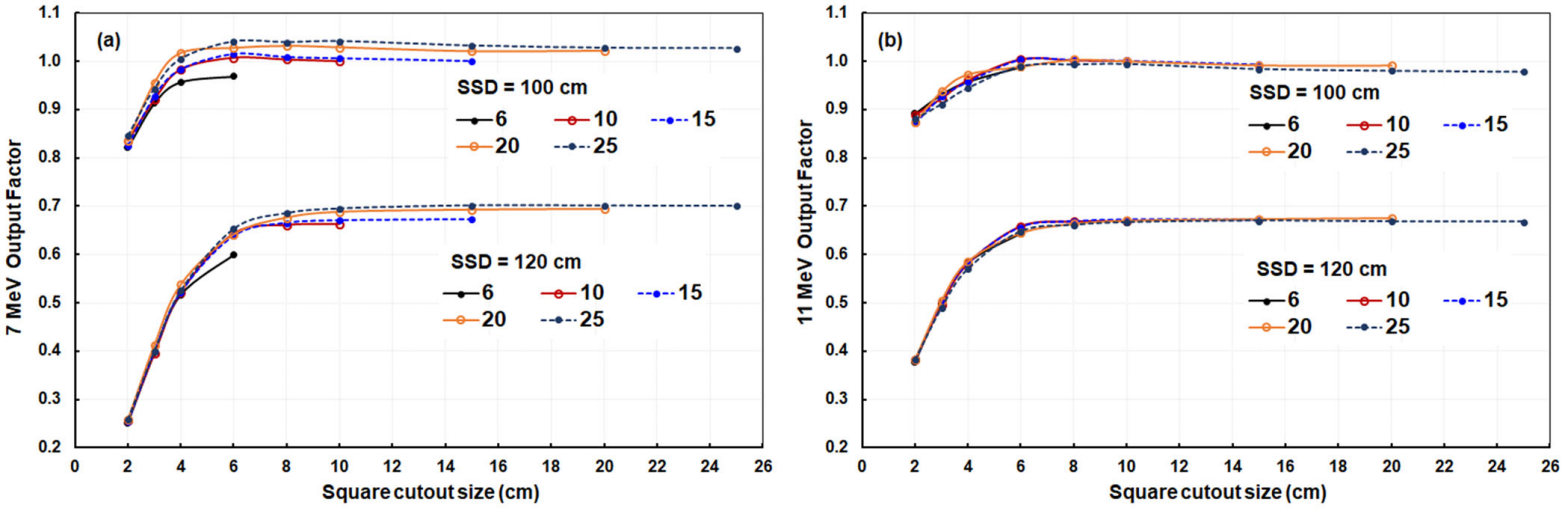
The output factors of (a) 7‐MeV and (b) 11‐MeV beams for various cone sizes of 6, 10, 15, 20, and 25 cm^2^ at SSDs of 100 and 120 cm. SSDs, source‐to‐surface distances

The variation in OFs for 7‐ and 11‐MeV beams with various field sizes of cone/cutout combinations showed substantial increases in OFs with field size until the field size neared 5 × 5 cm^2^ (Figure 3). These field sizes closely correspond to the minimum field sizes required for electronic equilibrium for different beam energies (i.e., 4.5 × 4.5 cm^2^ for the 7 MeV and 5.3 × 5.3 cm^2^ for the 11 MeV).^6^ The ratios of the OFs of all measured points between the two linacs were 1.000 ± 0.007 for the 7‐MeV beam and 1.004 ± 0.007 for the 11‐MeV beam, which indicated excellent matching in the OFs of both linacs.

### 3.3 TPS model validation

Measured PDDs and profiles were compared with TPS calculations with standard cones at 100‐cm SSD. For beam profiles, all point‐to‐point differences were within 3% in the high‐ and low‐dose regions, and the DTAs were within 3 mm in the penumbra region (Table 4). For PDDs, except for a few points near the surface of the PDD curves, all other point‐to‐point differences in the high‐ and low‐dose regions were within 3%; the maximum point‐to‐point difference in the point near the surface was 3.2% for the 11‐MeV beam. The DTAs in the deep‐falloff high‐gradient region were within 3 mm (Table 4). These results meet the MPPG 5.a criteria.

**TABLE 4:** Maximum point‐to‐point dose difference (%) and DTA (mm) of TPS calculation versus measurement from PDD curves and beam profiles at SSD = 100 cm

**7 MeV**	**PDD**				**Beam profile *d* = 1.66** **cm**	
Cone (cm^2^)	High‐dose region	Low‐dose region	High‐gradient region DTA (mm)	High‐dose region	Low‐dose region	Penumbra DTA (mm)
6	1.8	−0.5	0.7	−1.8	1.9	1.7
10	1.8	−0.5	0.5	−1.8	1.7	1.9
15	2.0	0.2	0.8	−2.0	1.6	2.0
20	2.5	0.2	1.5	2.5	2.2	1.8
25	2.9	0.2	1.3	2.7	2.0	2.1

**11 MeV**	**PDD**			**Beam profile *d* = 2.0** **cm**		
Cone (cm^2^)	High‐dose region	Low‐dose region	High‐gradient region DTA (mm)	High‐dose region	Low‐dose region	Penumbra DTA (mm)
6	2.5	0.5	0.4	1.6	1.6	1.5
10	2.5	0.5	0.4	−0.8	2.0	1.5
15	2.0	0.4	0.2	−2.3	1.3	2.5
20	3.2	0.4	0.5	1.9	1.6	1.7
25	2.7	0.3	0.4	1.6	1.6	2.0

Abbreviations: DTA, distance‐to‐agreement; PDD, percent depth dose; SSD, source‐to‐surface distance, TPS, treatment planning system.

The percent differences between the output data measured for optimal cone/cutout combinations and the TPS‐calculated dose per monitor unit (cGy/MU) were −0.1% ± 1.0% (max: 1.8%, min: −2.9%) for the 7‐MeV beam and 0.2% ± 0.8% (max: 2.6%, min: −1.2%) for the 11‐MeV beam (Table 5). All these results were within 3% for these optimal cone/cutout combinations. The optimal cone/cutout ratios [1.3, 2.0] were obtained from the comparison of a large set of cone/cutout combinations (described further in Section 4).

**TABLE 5:** Output factor differences (%) of TPS calculation versus measurement for optimal cone/cutout (cm^2^) combinations at different SSDs (cm)

**Cone**	**6 × 6**		**10 × 10**		**15 × 15**			**20 × 20**			**25 × 25**		
**Cutout**	4	**6**	5	**10**	8	4 × 12	**15**	12	6 × 18	**20**	**18**	8 × 24	**25**
**SSD (cm)**	**7 MeV**												
100	1.8	−0.2	−0.7	0.0	−0.1	0.4	−0.6	0.2	0.2	0.4	0.8	0.9	0.4
105	0.7	−1.1	−1.5	−0.4	−0.2	0.1	−0.3	0.0	0.2	−0.2	0.7	0.7	1.2
110	0.0	−1.6	−1.2	−1.0	0.6	0.1	−0.3	0.3	0.3	0.3	0.7	1.2	0.7
115	−1.2	−1.9	−2.3	−0.8	1.3	−1.1	−0.7	0.1	0.8	0.2	0.2	1.4	0.9
120	−2.9	−2.2	−2.9	−0.7	1.3	−1.2	−1.1	0.2	1.2	0.1	0.3	1.6	0.8
**SSD (cm)**	**11 MeV**												
100	1.2	−0.4	0.1	0.0	−0.1	1.1	−0.3	1.3	0.9	0.4	1.0	1.1	0.5
105	0.8	−0.6	−0.6	−0.8	0.2	1.1	−0.1	0.6	0.5	0.2	0.5	0.9	0.5
110	0.7	−0.6	0.2	−1.2	0.2	0.9	−0.5	0.5	0.8	−0.1	0.6	1.0	0.5
115	2.6	−0.5	−0.9	−1.1	0.4	1.0	−0.4	0.1	0.8	0.4	0.6	0.9	0.2
120	−1.2	−0.7	−1.0	−1.1	0.7	−0.4	−0.9	0.6	1.1	0.5	−0.1	1.7	0.3

The 12 × 12, 18 × 18 cm^2^, and rectangular field–measured data are interpolated.

Abbreviations: SSDs, source‐to‐surface distances; TPS, treatment planning system.

For the oblique beam incidence comparison, the point‐to‐point dose differences (%) from beam profiles were within 5% in the high‐ and low‐dose regions and the DTAs in the penumbra region were within 3 mm between TPS calculations and measurements. The measured and TPS‐calculated point dose differences were within 5% in the high‐dose region, and the DTAs in the high‐gradient region were within 3 mm (Table 6). All these results meet the MPPG 5.a criteria.

**TABLE 6:** Maximum point‐to‐point dose difference (%) in high‐ and low‐dose regions and the DTA (mm) in high‐gradient region of TPS calculation versus measurement from beam profiles and point dose measurements at the oblique beam incidence

		Beam profile			Point dose	
Energy (MeV)	Depth (cm)	High‐dose region	Low‐dose region	DTA (mm)	High‐dose region	DTA (mm)
7	1.6	1.5	2.7	2.6	1.0	–
	2.9	3.8	2.3	2.8	–	1.6
11	2.4	3.3	4.1	1.9	0.6	–
	4.3	−3.2	4.0	1.8	–	2.5

SSD = 100 cm, 10 × 10 cm^2^ cone, gantry angle 20°.

Abbreviations: DTA, distance‐to‐agreement; SSD, source‐to‐surface distance; TPS, treatment planning system.

### 3.4 Commissioning of RadCalc monitor unit calculation software

The effective SSD values (Table 7) from the measured data were used in the RadCalc software.

**TABLE 7:** The effective source‐to‐surface distance (cm) versus cone size

Cone (cm^2^)	7 MeV	11 MeV
6	72.1	81.5
10	86.2	87.6
15	89.6	89.9
20	92.1	92.0
25	93.5	92.7

For PDD validation, the RadCalc‐calculated dose rates (cGy/MU) for cutouts of 2 × 2, 3 × 3, 4 × 4, and 5 × 5 cm^2^ and five standard cones at depths from 1.0 to 3.6 cm for the 7‐MeV beam and from 1.0 to 5.0 cm for the 11‐MeV beam were compared with those calculated from the measured data. The differences were within 0.5% for both beams for all cone/cutout combinations.

The discrepancies between RadCalc‐calculated MUs and those calculated from the measured data were within 1.0% for all cone/cutout combinations except for the 2 × 4‐cm^2^ cutout that was within 3.0% at SSDs from 100 to 120 cm.

## 4 DISCUSSION

For the purpose of beam modeling for the RayStation TPS, we measured the open (without applicator) air fluence profiles in in‐ and cross‐plane axes and the air point fluence OF (OF_air_) at SDDs of 70 and 90 cm with an 8 × 8‐cm^2^ field and with rectangular fields of 20 × 8, 30 × 8, and 30 × 30 cm^2^ for each beam. The in‐air measurements were performed with a CC04 ionization chamber without a buildup cap in an empty 3D water scanning system. The OF_air_ is the measured signal that represented the relative electron fluence in air at the location of the detector. The OF_air_ is normalized relative to the 8 × 8‐cm^2^ field with an SSD of 70 cm (depths: 76 and 96 cm).

We validated the RayStation TPS calculation against the measurement data for a comprehensive set of cone/cutout combinations for SSDs varying from 100 to 120 cm. For the small cutout fields, 2 × 2, 3 × 3 and 2 × 4, 4 × 4 cm^2^, the TPS‐calculated OFs were different from measurements by more than 3%; generally, the discrepancy increases with increasing SSD. The results for 100 and 120‐cm SSDs are presented in Table 8. These results were similar for SSDs between 100 and 120 cm. The discrepancy increased with the SSDs because of the increasing air gap between the cutout and the surface. As we are aware, air gap significantly reduces the lateral beam uniformity for electron beams.^7^ After examining the differences between TPS calculations and the measured data, for the same cutout field size, we found that the discrepancy was also related to the ratio of cone and cutout sizes, *ratio =* *cone/cutout*; the optimal ratios were found to be [1.3, 2.0]. The field size of the rectangular field was calculated with the square‐root method.

**TABLE 8:** Differences in output factors (%) of TPS calculations versus measurements for various cone/cutout (cm^2^) combinations at different SSDs (cm)

Cutout	7 MeV, SSD = 100 cm					7 MeV, SSD = 120 cm				
Cone	6	10	15	20	25	6	10	15	20	25
2	1.6	2.1	1.0	1.0	2.1	−10.5	−10.0	−11.1	−10.5	−12.0
2 × 4	4.0	4.2	4.6	5.1	4.9	−5.7	−5.9	−5.9	−4.2	−7.1
3	2.1	0.8	2.5	4.3	3.0	−9.4	−11.6	−10.2	−7.8	−11.8
4	1.8	0.2	1.0	3.3	2.1	−2.9	−5.1	−4.7	−2.0	−5.5
5	−0.9	−0.7	0.0	1.1	1.6	−6.5	−2.9	−3.0	−2.8	−3.6
6	−0.2	0.2	0.8	1.2	2.5	−2.2	1.2	1.5	0.4	2.2
8		−0.6	−0.1	0.9	1.6		−0.3	1.3	1.6	2.9
10		0.0	0.1	0.2	1.3		−0.7	−0.6	1.7	2.6
12			−0.2	0.2	1.7			−1.4	0.2	1.5
15			−0.6	−0.4	0.9			−1.1	−0.2	1.9
4 × 12			0.4	2.3	2.1			−1.2	−0.2	−1.2
18				0.2	0.8				−0.2	0.3
20				0.4	0.4				0.1	0.7
6 × 18				0.2	1.7				1.2	2.0
25					0.4					0.8
8 × 24					0.9					1.6

Cutout	11 MeV, SSD = 100 cm					11 MeV, SSD = 120 cm				
2	3.8	3.9	2.3	3.2	4.8	−4.4	−3.4	−4.8	−3.5	−2.1
2 × 4	4.6	4.7	4.4	6.3	6.1	0.2	0.0	0.3	1.4	0.6
3	2.2	1.9	2.7	5.3	3.1	−4.6	−4.8	−4.2	−2.2	−4.7
4	1.2	1.4	1.9	5.1	3.3	−1.2	−0.4	−0.8	0.7	−1.1
5	−0.2	0.1	1.2	2.0	2.2	−3.2	−1.0	−1.1	−0.1	−0.8
6	−0.4	0.5	1.1	1.5	2.4	−0.7	1.5	1.8	0.7	2.2
8		−0.6	−0.1	1.1	1.6		−0.3	0.7	1.4	1.9
10		0.0	0.1	1.0	1.5		−1.1	−0.1	0.8	1.7
12			0.0	1.3	1.6			−1.0	0.6	1.2
15			−0.3	0.5	0.8			−0.9	0.1	1.2
4 × 12			1.1	0.5	2.2			−0.4	1.3	0.8
18				0.4	1.0				0.3	−0.1
20				0.9	0.8				0.5	0.7
6 × 18				0.9	1.8				1.1	1.7
25					0.5					0.3
8 × 24					1.1					1.7

Measured data from the 12 × 12, 18 × 18 cm^2^, and rectangular fields are interpolated.

Abbreviations: SSDs, source‐to‐surface distances; TPS, treatment planning system.

In every case where the disagreement was greater than 3%, the field sizes are too small for electronic equilibrium and are below the minimum recommended field sizes^6^ for these 7‐ and 11‐MeV beams. For small fields, Das et al. indicated that the target coverage could be increased by placing a cutout at the surface of the patient or by reducing the air gap between the electron applicator/cone and the surface.^7^ The findings from the present study of the 7‐ and 11‐MeV beams are consistent with these previous results.

As implementing these beams in the clinic, we have monitored their stability. Monthly QA for 23 months for one machine and 18 months for the other machine indicated that the differences in beam flatness from the baseline were −0.1% ± 0.2% (max: 0.5%, min: −0.6%) for 7 MeV and −0.1% ± 0.1% (max: 0.3%, min: −0.4%) for 11 MeV; the differences of beam energy from the baseline were: 0.2% ± 0.6% (max: 1.1%, min: −0.9%) for 7 MeV and 0.2% ± 0.8% (max: 0.8%, min: −0.6%) for 11 MeV. The variations in energy are much less than 1.0 mm in *R*_80_ and *R*_50_. Our long‐term periodic QA results demonstrated that beam characteristic parameters, such as output flatness, symmetry, and energy behave similarly to those of the standard electron beams.

## 5 CONCLUSIONS

By using the ICP/DW system, we were able to implement two new electron energies on our TrueBeam platform in a relatively short time (a few hours per beam) and to achieve highly consistent results across two linacs. Validation of the machine commissioning and TPS beam models indicate that all dosimetry characteristic parameters meet the MPPG 5.a criteria. The electron energies for these two linacs are now 6, 7, 9, 11, 12, 16, and 20 MeV corresponding to *R*_90_ of 1.7, 2.2, 2.7, 3.3, 3.8, 5.0, and 5.9 cm, respectively. The spacing of the *R*_90_ (and *R*_80_) from 6‐ to 12‐MeV beams is now 0.5 cm.

## CONFLICTS OF INTEREST

None.

## AUTHOR CONTRIBUTIONS

Rajat J. Kudchadker, Peter A. Balter, Song Gao: designed the study and edited the manuscript.

Song Gao, Manickam Muruganandham, Weiliang Du: collected beam commissioning data, and analyzed results, and edited this manuscript.

Song Gao, wrote the manuscript, RadCalc software commissioning.

Jared Ohrt: RayStation TPS modeling and MPPG 5.a testing.

## Supporting information

Supporting Information

Supporting Information
