# Supplementary material for: Adding customized electron energy beams to TrueBeam linear accelerators
Source: J Appl Clin Med Phys. 2022 May 9;23(7):e13633. doi: 10.1002/acm2.13633 (PMC9278672; doi:10.1002/acm2.13633)
Supplement: Supplementary file 1 — Supporting Information [file ACM2-23-e13633-s001.docx]

**Supplemental 1. The PDD values and plots versus field size of 7- and 11- MeV beams**

| **7 MeV Percentage Depth Dose (PDD), SSD = 100 cm** | | | | | | | | | | |
| --- | --- | --- | --- | --- | --- | --- | --- | --- | --- | --- |
| **depth (cm)** | **cone 6x6** | | | **cone 10x10** | | **Cone 10x10** | | **Cone 15x15** | **Cone 20x20** | **Cone 25x25** |
|  | **2x2** | **3x3** | **4x4** | **5x5** | **6x6** | **8x8** | **10x10** |  |  |  |
| **0.0** | 92.2 | 84.5 | 80.2 | 79.2 | 79.0 | 79.5 | 79.7 | 80.1 | 80.8 | 81.4 |
| **0.1** | 93.3 | 85.7 | 81.3 | 80.3 | 80.1 | 80.7 | 80.9 | 81.3 | 82.0 | 82.5 |
| **0.2** | 94.6 | 87.1 | 82.7 | 81.7 | 81.5 | 82.0 | 82.3 | 82.6 | 83.2 | 83.7 |
| **0.3** | 95.8 | 88.5 | 84.1 | 83.2 | 82.9 | 83.4 | 83.7 | 83.9 | 84.6 | 85.0 |
| **0.4** | 97.0 | 90.0 | 85.6 | 84.7 | 84.4 | 84.9 | 85.1 | 85.3 | 85.9 | 86.3 |
| **0.5** | 98.0 | 91.4 | 87.1 | 86.2 | 85.8 | 86.3 | 86.6 | 86.7 | 87.2 | 87.6 |
| **0.6** | 98.9 | 92.8 | 88.6 | 87.7 | 87.4 | 87.8 | 88.0 | 88.2 | 88.6 | 89.0 |
| **0.7** | 99.5 | 94.1 | 90.1 | 89.1 | 88.9 | 89.3 | 89.4 | 89.5 | 90.0 | 90.4 |
| **0.8** | 99.9 | 95.4 | 91.5 | 90.7 | 90.4 | 90.8 | 91.0 | 91.0 | 91.4 | 91.8 |
| **0.9** | 100.0 | 96.6 | 93.0 | 92.2 | 92.0 | 92.3 | 92.5 | 92.5 | 92.8 | 93.2 |
| **1.0** | 99.8 | 97.7 | 94.5 | 93.8 | 93.5 | 93.8 | 93.9 | 94.0 | 94.2 | 94.6 |
| **1.1** | 99.3 | 98.7 | 95.8 | 95.2 | 95.0 | 95.3 | 95.4 | 95.4 | 95.6 | 95.9 |
| **1.2** | 98.3 | 99.4 | 97.1 | 96.6 | 96.4 | 96.7 | 96.7 | 96.7 | 96.9 | 97.1 |
| **1.3** | 96.8 | 99.9 | 98.3 | 97.8 | 97.7 | 97.9 | 98.0 | 97.9 | 98.1 | 98.2 |
| **1.4** | 94.9 | 100.0 | 99.1 | 98.9 | 98.7 | 98.9 | 98.9 | 98.9 | 99.0 | 99.1 |
| **1.5** | 92.6 | 99.8 | 99.8 | 99.7 | 99.6 | 99.6 | 99.7 | 99.6 | 99.7 | 99.8 |
| **1.6** | 89.8 | 99.1 | 100.0 | 100.0 | 100.0 | 100.0 | 100.0 | 100.0 | 100.0 | 100.0 |
| **1.7** | 86.7 | 98.0 | 99.7 | 100.0 | 100.0 | 99.9 | 99.9 | 99.9 | 99.9 | 99.8 |
| **1.8** | 83.3 | 96.4 | 99.0 | 99.4 | 99.5 | 99.3 | 99.3 | 99.3 | 99.2 | 99.2 |
| **1.9** | 79.7 | 94.3 | 97.8 | 98.4 | 98.4 | 98.2 | 98.2 | 98.2 | 98.1 | 97.9 |
| **2.0** | 75.6 | 91.6 | 95.9 | 96.6 | 96.8 | 96.4 | 96.4 | 96.5 | 96.2 | 96.1 |
| **2.1** | 71.6 | 88.6 | 93.6 | 94.3 | 94.4 | 94.1 | 94.0 | 94.1 | 93.8 | 93.6 |
| **2.2** | 67.4 | 85.0 | 90.5 | 91.4 | 91.4 | 91.0 | 90.9 | 91.0 | 90.8 | 90.5 |
| **2.3** | 63.1 | 81.0 | 86.8 | 87.8 | 87.7 | 87.3 | 87.2 | 87.3 | 87.0 | 86.7 |
| **2.4** | 58.7 | 76.5 | 82.4 | 83.5 | 83.4 | 82.8 | 82.7 | 82.9 | 82.5 | 82.3 |
| **2.5** | 54.5 | 71.7 | 77.8 | 78.7 | 78.5 | 78.1 | 77.9 | 78.1 | 77.6 | 77.4 |
| **2.6** | 50.1 | 66.6 | 72.1 | 73.2 | 72.8 | 72.4 | 72.1 | 72.4 | 72.1 | 71.8 |
| **2.7** | 45.6 | 60.9 | 66.2 | 67.2 | 66.8 | 66.3 | 66.0 | 66.4 | 65.8 | 65.7 |
| **2.8** | 41.1 | 55.2 | 59.8 | 60.7 | 60.3 | 59.8 | 59.6 | 59.9 | 59.5 | 59.2 |
| **2.9** | 36.7 | 49.4 | 53.3 | 54.3 | 53.6 | 53.0 | 52.9 | 53.2 | 52.8 | 52.6 |
| **3.0** | 32.2 | 43.2 | 46.4 | 47.0 | 46.5 | 46.0 | 45.9 | 46.2 | 45.9 | 45.6 |
| **3.1** | 27.8 | 37.1 | 39.4 | 39.9 | 39.4 | 39.2 | 39.1 | 39.4 | 39.2 | 38.6 |
| **3.2** | 23.6 | 31.1 | 33.1 | 33.4 | 32.9 | 32.6 | 32.4 | 32.8 | 32.5 | 32.3 |
| **3.3** | 19.5 | 25.4 | 26.6 | 27.0 | 26.4 | 26.2 | 26.0 | 26.3 | 26.2 | 26.0 |
| **3.4** | 15.5 | 20.0 | 20.9 | 21.2 | 20.7 | 20.5 | 20.4 | 20.7 | 20.4 | 20.4 |
| **3.5** | 12.2 | 15.4 | 15.9 | 16.2 | 15.7 | 15.5 | 15.5 | 15.7 | 15.5 | 15.5 |
| **3.6** | 9.2 | 11.4 | 11.7 | 12.0 | 11.5 | 11.4 | 11.3 | 11.5 | 11.4 | 11.3 |
| **3.7** | 6.7 | 8.1 | 8.3 | 8.4 | 8.1 | 8.1 | 8.0 | 8.1 | 8.1 | 8.0 |
| **3.8** | 4.8 | 5.6 | 5.6 | 5.7 | 5.6 | 5.5 | 5.5 | 5.5 | 5.5 | 5.5 |
| **3.9** | 3.4 | 3.8 | 3.8 | 3.9 | 3.7 | 3.8 | 3.7 | 3.8 | 3.8 | 3.7 |
| **4.0** | 2.4 | 2.6 | 2.5 | 2.7 | 2.5 | 2.5 | 2.5 | 2.5 | 2.5 | 2.5 |
| **4.1** | 1.5 | 1.8 | 1.8 | 1.9 | 1.8 | 1.8 | 1.8 | 1.8 | 1.8 | 1.8 |
| **4.2** | 1.2 | 1.4 | 1.4 | 1.5 | 1.4 | 1.4 | 1.4 | 1.4 | 1.4 | 1.4 |
| **4.3** | 1.0 | 1.2 | 1.1 | 1.2 | 1.1 | 1.2 | 1.2 | 1.2 | 1.2 | 1.2 |
| **4.4** | 0.9 | 0.9 | 1.1 | 1.1 | 1.1 | 1.1 | 1.1 | 1.1 | 1.1 | 1.1 |
| **4.5** | 0.9 | 0.9 | 0.9 | 1.0 | 0.9 | 0.9 | 0.9 | 0.9 | 0.9 | 0.9 |
| **4.6** | 0.9 | 0.9 | 0.9 | 0.9 | 0.9 | 0.9 | 0.9 | 0.9 | 0.9 | 0.9 |
| **4.7** | 0.9 | 0.9 | 0.9 | 0.9 | 0.9 | 0.9 | 0.9 | 0.9 | 0.9 | 0.9 |
| **4.8** | 0.8 | 0.8 | 0.8 | 0.9 | 0.9 | 0.9 | 0.9 | 0.9 | 0.9 | 0.9 |
| **4.9** | 0.8 | 0.8 | 0.8 | 0.9 | 0.8 | 0.9 | 0.9 | 0.9 | 0.9 | 0.9 |
| **5.0** | 0.8 | 0.8 | 0.8 | 0.9 | 0.8 | 0.9 | 0.9 | 0.9 | 0.9 | 0.9 |
| **5.1** | 0.8 | 0.8 | 0.8 | 0.9 | 0.8 | 0.9 | 0.9 | 0.9 | 0.9 | 0.9 |
| **5.2** | 0.8 | 0.8 | 0.8 | 0.9 | 0.8 | 0.9 | 0.8 | 0.9 | 0.9 | 0.9 |
| **5.3** | 0.8 | 0.8 | 0.8 | 0.8 | 0.8 | 0.9 | 0.8 | 0.9 | 0.9 | 0.8 |
| **5.4** | 0.8 | 0.8 | 0.8 | 0.8 | 0.8 | 0.9 | 0.8 | 0.8 | 0.9 | 0.8 |
| **5.5** | 0.8 | 0.8 | 0.8 | 0.8 | 0.8 | 0.8 | 0.8 | 0.8 | 0.9 | 0.8 |
| **5.6** | 0.8 | 0.8 | 0.8 | 0.8 | 0.8 | 0.9 | 0.8 | 0.8 | 0.8 | 0.8 |
| **5.7** | 0.8 | 0.8 | 0.8 | 0.8 | 0.8 | 0.8 | 0.8 | 0.8 | 0.8 | 0.8 |
| **5.8** | 0.8 | 0.8 | 0.8 | 0.8 | 0.8 | 0.8 | 0.8 | 0.8 | 0.8 | 0.8 |
| **5.9** | 0.8 | 0.8 | 0.8 | 0.8 | 0.8 | 0.8 | 0.8 | 0.8 | 0.8 | 0.8 |
| **6.0** | 0.8 | 0.7 | 0.7 | 0.8 | 0.8 | 0.8 | 0.8 | 0.8 | 0.8 | 0.8 |
| **6.1** | 0.7 | 0.7 | 0.7 | 0.8 | 0.7 | 0.8 | 0.8 | 0.8 | 0.8 | 0.8 |
| **6.2** | 0.7 | 0.7 | 0.7 | 0.8 | 0.7 | 0.8 | 0.8 | 0.8 | 0.8 | 0.8 |
| **6.3** | 0.7 | 0.7 | 0.7 | 0.8 | 0.7 | 0.8 | 0.8 | 0.8 | 0.8 | 0.8 |
| **6.4** | 0.7 | 0.7 | 0.7 | 0.8 | 0.7 | 0.8 | 0.8 | 0.8 | 0.8 | 0.8 |
| **6.5** | 0.7 | 0.7 | 0.7 | 0.8 | 0.7 | 0.8 | 0.8 | 0.8 | 0.8 | 0.8 |
| **6.6** | 0.7 | 0.7 | 0.7 | 0.8 | 0.7 | 0.8 | 0.8 | 0.8 | 0.8 | 0.8 |
| **6.7** | 0.7 | 0.7 | 0.7 | 0.8 | 0.7 | 0.8 | 0.8 | 0.7 | 0.8 | 0.8 |
| **6.8** | 0.7 | 0.7 | 0.7 | 0.8 | 0.7 | 0.8 | 0.8 | 0.7 | 0.8 | 0.8 |
| **6.9** | 0.7 | 0.7 | 0.7 | 0.7 | 0.7 | 0.7 | 0.8 | 0.7 | 0.8 | 0.8 |
| **7.0** | 0.7 | 0.7 | 0.7 | 0.7 | 0.7 | 0.7 | 0.7 | 0.7 | 0.8 | 0.8 |
| **7.1** | 0.7 | 0.7 | 0.7 | 0.7 | 0.7 | 0.7 | 0.7 | 0.7 | 0.8 | 0.7 |
| **7.2** | 0.7 | 0.7 | 0.7 | 0.7 | 0.7 | 0.7 | 0.7 | 0.7 | 0.8 | 0.7 |
| **7.3** | 0.7 | 0.6 | 0.7 | 0.7 | 0.7 | 0.7 | 0.7 | 0.7 | 0.7 | 0.7 |
| **7.4** | 0.7 | 0.6 | 0.7 | 0.7 | 0.7 | 0.7 | 0.7 | 0.7 | 0.7 | 0.7 |
| **7.5** | 0.7 | 0.6 | 0.6 | 0.7 | 0.7 | 0.7 | 0.7 | 0.7 | 0.7 | 0.7 |
| **7.6** | 0.6 | 0.6 | 0.6 | 0.7 | 0.7 | 0.7 | 0.7 | 0.7 | 0.7 | 0.7 |
| **7.7** | 0.7 | 0.6 | 0.6 | 0.7 | 0.6 | 0.7 | 0.7 | 0.7 | 0.7 | 0.7 |
| **7.8** | 0.6 | 0.6 | 0.6 | 0.7 | 0.6 | 0.7 | 0.7 | 0.7 | 0.7 | 0.7 |
| **7.9** | 0.6 | 0.6 | 0.6 | 0.7 | 0.6 | 0.7 | 0.7 | 0.7 | 0.7 | 0.7 |
| **8.0** | 0.6 | 0.6 | 0.6 | 0.7 | 0.6 | 0.7 | 0.7 | 0.7 | 0.7 | 0.7 |

| **11 MeV Percentage Depth Dose (PDD), SSD = 100 cm** | | | | | | | | | | |
| --- | --- | --- | --- | --- | --- | --- | --- | --- | --- | --- |
| **depth (cm)** | **Cone 6x6** | | | **Cone 10x10** | | **Cone 10x10** | | **15x15** | **20x20** | **25x25** |
|  | **2x2** | **3x3** | **4x4** | **5x5** | **6x6** | **8x8** | **10x10** |  |  |  |
| **0.0** | 93.9 | 89.6 | 86.5 | 84.9 | 84.0 | 84.1 | 84.1 | 84.1 | 84.7 | 85.0 |
| **0.1** | 94.7 | 90.5 | 87.4 | 85.7 | 84.8 | 85.0 | 85.2 | 85.0 | 85.8 | 85.8 |
| **0.2** | 95.7 | 91.5 | 88.5 | 86.8 | 85.8 | 86.0 | 86.2 | 85.9 | 86.7 | 86.7 |
| **0.3** | 96.7 | 92.6 | 89.5 | 87.9 | 86.8 | 86.9 | 87.1 | 86.9 | 87.6 | 87.6 |
| **0.4** | 97.6 | 93.6 | 90.5 | 88.8 | 87.7 | 87.8 | 88.1 | 87.8 | 88.5 | 88.5 |
| **0.5** | 98.3 | 94.5 | 91.4 | 89.6 | 88.6 | 88.7 | 88.9 | 88.6 | 89.3 | 89.2 |
| **0.6** | 98.9 | 95.3 | 92.1 | 90.4 | 89.4 | 89.4 | 89.6 | 89.4 | 90.0 | 89.9 |
| **0.7** | 99.3 | 96.0 | 92.9 | 91.2 | 90.1 | 90.2 | 90.3 | 90.1 | 90.7 | 90.6 |
| **0.8** | 99.7 | 96.7 | 93.6 | 92.0 | 90.8 | 90.9 | 91.0 | 90.8 | 91.3 | 91.3 |
| **0.9** | 99.9 | 97.3 | 94.2 | 92.6 | 91.5 | 91.5 | 91.7 | 91.5 | 92.0 | 91.9 |
| **1.0** | 100.0 | 97.9 | 94.9 | 93.2 | 92.2 | 92.2 | 92.3 | 92.2 | 92.6 | 92.5 |
| **1.1** | 100.0 | 98.4 | 95.5 | 93.9 | 92.8 | 92.8 | 93.0 | 92.8 | 93.2 | 93.2 |
| **1.2** | 99.8 | 98.8 | 96.1 | 94.6 | 93.5 | 93.4 | 93.6 | 93.5 | 93.8 | 93.8 |
| **1.3** | 99.4 | 99.2 | 96.7 | 95.2 | 94.2 | 94.1 | 94.3 | 94.1 | 94.5 | 94.4 |
| **1.4** | 98.9 | 99.6 | 97.3 | 95.8 | 94.8 | 94.8 | 94.9 | 94.8 | 95.1 | 95.1 |
| **1.5** | 98.2 | 99.8 | 97.9 | 96.4 | 95.5 | 95.4 | 95.6 | 95.4 | 95.7 | 95.7 |
| **1.6** | 97.2 | 100.0 | 98.4 | 97.0 | 96.2 | 96.1 | 96.2 | 96.1 | 96.4 | 96.4 |
| **1.7** | 95.9 | 100.0 | 98.8 | 97.6 | 96.9 | 96.8 | 96.8 | 96.8 | 97.0 | 97.0 |
| **1.8** | 94.4 | 99.9 | 99.2 | 98.2 | 97.5 | 97.4 | 97.5 | 97.4 | 97.6 | 97.6 |
| **1.9** | 92.6 | 99.7 | 99.6 | 98.7 | 98.1 | 98.0 | 98.1 | 98.1 | 98.2 | 98.2 |
| **2.0** | 90.6 | 99.2 | 99.8 | 99.2 | 98.7 | 98.6 | 98.7 | 98.6 | 98.7 | 98.7 |
| **2.1** | 88.3 | 98.6 | 100.0 | 99.6 | 99.2 | 99.1 | 99.1 | 99.1 | 99.2 | 99.2 |
| **2.2** | 85.8 | 97.7 | 100.0 | 99.8 | 99.6 | 99.5 | 99.5 | 99.5 | 99.6 | 99.6 |
| **2.3** | 83.1 | 96.7 | 99.9 | 100.0 | 99.9 | 99.8 | 99.8 | 99.8 | 99.9 | 99.9 |
| **2.4** | 80.2 | 95.4 | 99.6 | 100.0 | 100.0 | 100.0 | 100.0 | 100.0 | 100.0 | 100.0 |
| **2.5** | 77.1 | 93.9 | 99.0 | 99.9 | 100.0 | 100.0 | 100.0 | 100.0 | 100.0 | 100.0 |
| **2.6** | 74.1 | 92.1 | 98.2 | 99.5 | 99.8 | 99.8 | 99.7 | 99.8 | 99.7 | 99.7 |
| **2.7** | 70.9 | 90.1 | 97.2 | 98.9 | 99.4 | 99.4 | 99.3 | 99.4 | 99.3 | 99.3 |
| **2.8** | 67.8 | 87.9 | 95.9 | 98.1 | 98.8 | 98.7 | 98.7 | 98.7 | 98.7 | 98.6 |
| **2.9** | 64.5 | 85.5 | 94.4 | 97.0 | 97.8 | 97.7 | 97.7 | 97.7 | 97.7 | 97.7 |
| **3.0** | 61.2 | 82.8 | 92.7 | 95.7 | 96.6 | 96.5 | 96.4 | 96.5 | 96.4 | 96.4 |
| **3.1** | 58.0 | 80.1 | 90.7 | 93.9 | 94.9 | 94.9 | 94.8 | 94.9 | 94.9 | 94.9 |
| **3.2** | 54.8 | 77.1 | 88.3 | 91.8 | 93.0 | 92.8 | 92.8 | 92.9 | 92.8 | 92.9 |
| **3.3** | 51.8 | 74.1 | 85.7 | 89.5 | 90.7 | 90.5 | 90.4 | 90.6 | 90.5 | 90.5 |
| **3.4** | 48.8 | 70.9 | 82.8 | 86.8 | 88.0 | 87.9 | 87.8 | 87.9 | 87.8 | 87.9 |
| **3.5** | 46.0 | 67.7 | 79.8 | 83.9 | 85.0 | 84.9 | 84.8 | 85.0 | 84.8 | 84.9 |
| **3.6** | 43.1 | 64.2 | 76.3 | 80.4 | 81.6 | 81.4 | 81.2 | 81.5 | 81.3 | 81.4 |
| **3.7** | 40.3 | 60.7 | 72.7 | 76.7 | 77.9 | 77.7 | 77.4 | 77.7 | 77.6 | 77.7 |
| **3.8** | 37.6 | 57.2 | 68.8 | 72.6 | 73.6 | 73.4 | 73.3 | 73.5 | 73.5 | 73.5 |
| **3.9** | 34.9 | 53.4 | 64.5 | 68.4 | 69.3 | 68.9 | 68.7 | 69.0 | 68.9 | 68.9 |
| **4.0** | 32.3 | 49.7 | 60.2 | 63.7 | 64.5 | 64.2 | 63.9 | 64.3 | 64.3 | 64.3 |
| **4.1** | 29.7 | 46.0 | 55.7 | 58.9 | 59.5 | 59.3 | 59.0 | 59.3 | 59.3 | 59.3 |
| **4.2** | 27.2 | 42.2 | 51.0 | 54.1 | 54.4 | 54.2 | 54.0 | 54.3 | 54.2 | 54.2 |
| **4.3** | 24.7 | 38.3 | 46.3 | 48.8 | 49.1 | 48.8 | 48.7 | 49.0 | 48.9 | 49.0 |
| **4.4** | 22.3 | 34.5 | 41.5 | 43.5 | 43.9 | 43.5 | 43.4 | 43.7 | 43.7 | 43.7 |
| **4.5** | 19.9 | 30.6 | 36.8 | 38.5 | 38.5 | 38.3 | 38.2 | 38.4 | 38.4 | 38.5 |
| **4.6** | 17.5 | 26.8 | 32.0 | 33.5 | 33.4 | 33.0 | 32.8 | 33.1 | 33.2 | 33.2 |
| **4.7** | 15.4 | 23.2 | 27.4 | 28.7 | 28.4 | 28.2 | 28.0 | 28.3 | 28.4 | 28.4 |
| **4.8** | 13.2 | 19.6 | 23.1 | 24.1 | 23.7 | 23.7 | 23.4 | 23.6 | 23.7 | 23.8 |
| **4.9** | 11.1 | 16.4 | 19.1 | 20.0 | 19.5 | 19.4 | 19.2 | 19.5 | 19.6 | 19.6 |
| **5.0** | 8.4 | 13.5 | 15.4 | 16.1 | 15.7 | 15.6 | 15.4 | 15.7 | 15.7 | 15.7 |
| **5.1** | 6.7 | 10.8 | 12.2 | 12.7 | 12.4 | 12.4 | 12.2 | 12.3 | 12.4 | 12.4 |
| **5.2** | 5.4 | 8.6 | 9.6 | 9.9 | 9.5 | 9.6 | 9.5 | 9.5 | 9.6 | 9.6 |
| **5.3** | 4.3 | 6.6 | 7.3 | 7.6 | 7.3 | 7.3 | 7.2 | 7.2 | 7.3 | 7.2 |
| **5.4** | 3.4 | 5.1 | 5.5 | 5.8 | 5.4 | 5.5 | 5.4 | 5.4 | 5.5 | 5.5 |
| **5.5** | 2.7 | 3.9 | 4.2 | 4.4 | 4.1 | 4.2 | 4.1 | 4.1 | 4.1 | 4.1 |
| **5.6** | 2.2 | 3.0 | 3.2 | 3.4 | 3.1 | 3.2 | 3.2 | 3.1 | 3.2 | 3.2 |
| **5.7** | 1.8 | 2.4 | 2.5 | 2.7 | 2.5 | 2.6 | 2.5 | 2.5 | 2.5 | 2.5 |
| **5.8** | 1.6 | 1.8 | 2.1 | 2.2 | 2.1 | 2.2 | 2.1 | 2.1 | 2.1 | 2.1 |
| **5.9** | 1.4 | 1.6 | 1.8 | 2.0 | 1.8 | 1.9 | 1.9 | 1.8 | 1.8 | 1.8 |
| **6.0** | 1.3 | 1.4 | 1.7 | 1.8 | 1.7 | 1.7 | 1.7 | 1.7 | 1.7 | 1.7 |
| **6.1** | 1.3 | 1.3 | 1.4 | 1.7 | 1.6 | 1.7 | 1.6 | 1.6 | 1.6 | 1.6 |
| **6.2** | 1.2 | 1.3 | 1.4 | 1.5 | 1.4 | 1.4 | 1.4 | 1.4 | 1.4 | 1.4 |
| **6.3** | 1.2 | 1.3 | 1.3 | 1.4 | 1.3 | 1.4 | 1.4 | 1.3 | 1.3 | 1.4 |
| **6.4** | 1.2 | 1.3 | 1.3 | 1.4 | 1.3 | 1.4 | 1.4 | 1.3 | 1.3 | 1.3 |
| **6.5** | 1.2 | 1.2 | 1.3 | 1.4 | 1.3 | 1.4 | 1.3 | 1.3 | 1.3 | 1.3 |
| **6.6** | 1.2 | 1.2 | 1.3 | 1.4 | 1.3 | 1.4 | 1.3 | 1.3 | 1.3 | 1.3 |
| **6.7** | 1.1 | 1.2 | 1.3 | 1.4 | 1.3 | 1.4 | 1.3 | 1.3 | 1.3 | 1.3 |
| **6.8** | 1.1 | 1.2 | 1.2 | 1.4 | 1.2 | 1.3 | 1.3 | 1.3 | 1.3 | 1.3 |
| **6.9** | 1.1 | 1.2 | 1.2 | 1.3 | 1.2 | 1.3 | 1.3 | 1.3 | 1.3 | 1.3 |
| **7.0** | 1.1 | 1.2 | 1.2 | 1.3 | 1.2 | 1.3 | 1.3 | 1.3 | 1.3 | 1.3 |
| **7.1** | 1.1 | 1.2 | 1.2 | 1.3 | 1.2 | 1.3 | 1.3 | 1.3 | 1.3 | 1.3 |
| **7.2** | 1.1 | 1.2 | 1.2 | 1.3 | 1.2 | 1.3 | 1.3 | 1.3 | 1.3 | 1.3 |
| **7.3** | 1.1 | 1.1 | 1.2 | 1.3 | 1.2 | 1.3 | 1.3 | 1.2 | 1.3 | 1.3 |
| **7.4** | 1.1 | 1.1 | 1.2 | 1.3 | 1.2 | 1.3 | 1.3 | 1.2 | 1.3 | 1.3 |
| **7.5** | 1.0 | 1.1 | 1.2 | 1.3 | 1.2 | 1.3 | 1.2 | 1.2 | 1.2 | 1.3 |
| **7.6** | 1.1 | 1.1 | 1.1 | 1.3 | 1.2 | 1.3 | 1.2 | 1.2 | 1.2 | 1.2 |
| **7.7** | 1.0 | 1.1 | 1.1 | 1.3 | 1.1 | 1.3 | 1.2 | 1.2 | 1.2 | 1.2 |
| **7.8** | 1.0 | 1.1 | 1.1 | 1.2 | 1.1 | 1.2 | 1.2 | 1.2 | 1.2 | 1.2 |
| **7.9** | 1.0 | 1.1 | 1.1 | 1.2 | 1.1 | 1.2 | 1.2 | 1.2 | 1.2 | 1.2 |
| **8.0** | 1.0 | 1.1 | 1.1 | 1.2 | 1.1 | 1.2 | 1.2 | 1.2 | 1.2 | 1.2 |
| **8.1** | 1.0 | 1.1 | 1.1 | 1.2 | 1.1 | 1.2 | 1.2 | 1.2 | 1.2 | 1.2 |
| **8.2** | 1.0 | 1.0 | 1.1 | 1.2 | 1.1 | 1.2 | 1.2 | 1.2 | 1.2 | 1.2 |
| **8.3** | 1.0 | 1.0 | 1.1 | 1.2 | 1.1 | 1.2 | 1.2 | 1.2 | 1.2 | 1.2 |
| **8.4** | 1.0 | 1.0 | 1.1 | 1.2 | 1.1 | 1.2 | 1.2 | 1.2 | 1.2 | 1.2 |
| **8.5** | 1.0 | 1.0 | 1.1 | 1.2 | 1.1 | 1.2 | 1.2 | 1.1 | 1.2 | 1.2 |
| **8.6** | 1.0 | 1.0 | 1.1 | 1.2 | 1.1 | 1.2 | 1.1 | 1.1 | 1.2 | 1.2 |
| **8.7** | 1.0 | 1.0 | 1.0 | 1.1 | 1.1 | 1.2 | 1.1 | 1.1 | 1.2 | 1.2 |
| **8.8** | 0.9 | 1.0 | 1.1 | 1.1 | 1.0 | 1.1 | 1.1 | 1.1 | 1.1 | 1.2 |
| **8.9** | 0.9 | 1.0 | 1.0 | 1.1 | 1.0 | 1.1 | 1.1 | 1.1 | 1.1 | 1.1 |
| **9.0** | 0.9 | 1.0 | 1.0 | 1.1 | 1.0 | 1.1 | 1.1 | 1.1 | 1.1 | 1.1 |
| **9.1** | 0.9 | 1.0 | 1.0 | 1.1 | 1.0 | 1.1 | 1.1 | 1.1 | 1.1 | 1.1 |
| **9.2** | 0.9 | 1.0 | 1.0 | 1.1 | 1.0 | 1.1 | 1.1 | 1.1 | 1.1 | 1.1 |
| **9.3** | 0.9 | 1.0 | 1.0 | 1.1 | 1.0 | 1.1 | 1.1 | 1.1 | 1.1 | 1.1 |
| **9.4** | 0.9 | 0.9 | 1.0 | 1.1 | 1.0 | 1.1 | 1.1 | 1.1 | 1.1 | 1.1 |
| **9.5** | 0.9 | 0.9 | 1.0 | 1.1 | 1.0 | 1.1 | 1.1 | 1.1 | 1.1 | 1.1 |
| **9.6** | 0.9 | 0.9 | 1.0 | 1.1 | 1.0 | 1.1 | 1.1 | 1.1 | 1.1 | 1.1 |
| **9.7** | 0.9 | 0.9 | 0.9 | 1.1 | 1.0 | 1.1 | 1.0 | 1.1 | 1.1 | 1.1 |
| **9.8** | 0.9 | 0.9 | 0.9 | 1.1 | 1.0 | 1.1 | 1.0 | 1.1 | 1.1 | 1.1 |
| **9.9** | 0.8 | 0.9 | 0.9 | 1.0 | 1.0 | 1.1 | 1.0 | 1.1 | 1.0 | 1.0 |
| **10.0** | 0.8 | 0.9 | 0.9 | 1.0 | 1.0 | 1.1 | 1.0 | 1.1 | 1.0 | 1.0 |
