# Supplementary material for: Adding customized electron energy beams to TrueBeam linear accelerators
Source: J Appl Clin Med Phys. 2022 May 9;23(7):e13633. doi: 10.1002/acm2.13633 (PMC9278672; doi:10.1002/acm2.13633)
Supplement: Supplementary file 2 — Supporting Information [file ACM2-23-e13633-s002.docx]

# Supplemental data 2. The 7-MeV and 11-MeV Beams Flatness and Symmetry

Flatness and symmetry were calculated from in-plane (radial) and cross-plane (transverse) scans using Sun Nuclear 3DS scanning system for different electron cones at an SSD of 100cm. Cone sizes: 6×6, 10×10, 15×15, 20×20, and 25×25 cm^2^ at three different depths (d_max_, R_90%_ and R_50%_).

| **7 MeV Inline: Flatness and Symmetry (%) Linac 1** | | | | | | | | | | |
| --- | --- | --- | --- | --- | --- | --- | --- | --- | --- | --- |
| **depth (cm)** | **Cone Size (cm^2^)** | | | | | | | | | |
|  | **6x6** | | **10x10** | | **15x15** | | **20x20** | | **25x25** | |
|  | **Flat** | **Symm** | **Flat** | **Symm** | **Flat** | **Symm** | **Flat** | **Symm** | **Flat** | **Symm** |
| **1.7** | 9.7 | 0.3 | 4.9 | 1.3 | 1.9 | 1.1 | 0.8 | 0.5 | 1.5 | 0.7 |
| **2.2** | 12.8 | 0.9 | 6.7 | 1.1 | 2.8 | 1.2 | 1.1 | 0.9 | 1.5 | 0.3 |
| **3.0** | 14.7 | 1.5 | 7.5 | 0.8 | 3.4 | 1.7 | 1.3 | 0.3 | 1.8 | 0.4 |
|  |  |  |  |  |  |  |  |  |  |  |
| **7 MeV Crossline: Flatness and Symmetry (%) Linac 1** | | | | | | | | | | |
| **depth (cm)** | **Cone Size (cm^2^)** | | | | | | | | | |
|  | **6x6** | | **10x10** | | **15x15** | | **20x20** | | **25x25** | |
|  | **Flat** | **Symm** | **Flat** | **Symm** | **Flat** | **Symm** | **Flat** | **Symm** | **Flat** | **Symm** |
| **1.7** | 9.8 | 0.4 | 4.7 | 0.9 | 2.1 | 0.5 | 0.9 | 0.4 | 1.1 | 0.2 |
| **2.2** | 12.9 | 0.7 | 6.4 | 0.6 | 2.8 | 0.4 | 1.0 | 0.4 | 1.2 | 0.4 |
| **3.0** | 14.7 | 1.3 | 7.3 | 0.3 | 3.0 | 0.6 | 1.6 | 1.4 | 2.0 | 1.6 |

| **7 MeV Inline: Flatness and Symmetry (%) Linac 2** | | | | | | | | | | |
| --- | --- | --- | --- | --- | --- | --- | --- | --- | --- | --- |
| **depth (cm)** | **Cone Size (cm^2^)** | | | | | | | | | |
|  | **6x6** | | **10x10** | | **15x15** | | **20x20** | | **25x25** | |
|  | **Flat** | **Symm** | **Flat** | **Symm** | **Flat** | **Symm** | **Flat** | **Symm** | **Flat** | **Symm** |
| **1.7** | 9.2 | 0.2 | 3.8 | 0.4 | 1.8 | 0.3 | 0.8 | 0.3 | 1.1 | 0.5 |
| **2.2** | 12.3 | 0.2 | 5.7 | 0.1 | 2.4 | 0.2 | 0.9 | 0.4 | 1.1 | 0.5 |
| **3.0** | 14.2 | 0.5 | 7.2 | 0.8 | 2.7 | 0.6 | 1.6 | 0.9 | 2.0 | 1.0 |
|  |  |  |  |  |  |  |  |  |  |  |
| **7 MeV Crossline: Flatness and Symmetry (%) Linac 2** | | | | | | | | | | |
| **depth (cm)** | **Cone Size (cm^2^)** | | | | | | | | | |
|  | **6x6** | | **10x10** | | **15x15** | | **20x20** | | **25x25** | |
|  | **Flat** | **Symm** | **Flat** | **Symm** | **Flat** | **Symm** | **Flat** | **Symm** | **Flat** | **Symm** |
| **1.7** | 9.2 | 0.1 | 4.5 | 1.5 | 1.7 | 0.9 | 0.8 | 0.8 | 1.3 | 0.7 |
| **2.2** | 12.3 | 0.2 | 6.4 | 1.4 | 2.5 | 1.1 | 1.0 | 0.7 | 1.4 | 0.9 |
| **3.0** | 14.2 | 0.6 | 7.5 | 1.4 | 3.0 | 1.8 | 1.6 | 1.4 | 2.2 | 1.2 |

| **11 MeV Inline: Flatness and Symmetry (%) Linac 1** | | | | | | | | | | |
| --- | --- | --- | --- | --- | --- | --- | --- | --- | --- | --- |
| **depth (cm)** | **Cone Size (cm^2^)** | | | | | | | | | |
|  | **6x6** | | **10x10** | | **15x15** | | **20x20** | | **25x25** | |
|  | **Flat** | **Symm** | **Flat** | **Symm** | **Flat** | **Symm** | **Flat** | **Symm** | **Flat** | **Symm** |
| **2.4** | 9.6 | 0.6 | 4.6 | 1.0 | 2.1 | 1.3 | 1.0 | 1.1 | 0.7 | 0.3 |
| **3.3** | 14.9 | 0.4 | 8.4 | 0.7 | 4.1 | 1.3 | 2.0 | 1.4 | 1.2 | 0.8 |
| **4.3** | 17.1 | 1.1 | 9.9 | 0.5 | 5.4 | 1.5 | 1.9 | 0.7 | 0.9 | 0.5 |
|  | | | | | | | | | | |
|  | | | | | | | | | | |
| **11 MeV Crossline: Flatness and Symmetry (%) Linac 1** | | | | | | | | | | |
| **depth (cm)** | **Cone Size (cm^2^)** | | | | | | | | | |
|  | **6x6** | | **10x10** | | **15x15** | | **20x20** | | **25x25** | |
|  | **Flat** | **Symm** | **Flat** | **Symm** | **Flat** | **Symm** | **Flat** | **Symm** | **Flat** | **Symm** |
| **2.4** | 9.6 | 0.8 | 4.4 | 0.5 | 2.2 | 0.5 | 0.8 | 0.2 | 0.8 | 0.3 |
| **3.3** | 14.9 | 0.4 | 8.2 | 0.1 | 4.1 | 0.4 | 1.6 | 0.2 | 1.0 | 0.1 |
| **4.3** | 17.2 | 1.1 | 9.8 | 0.3 | 4.9 | 0.6 | 1.8 | 0.6 | 1.1 | 0.5 |

| **11 MeV Inline: Flatness and Symmetry (%) Linac 2** | | | | | | | | | | |
| --- | --- | --- | --- | --- | --- | --- | --- | --- | --- | --- |
| **depth (cm)** | **Cone Size (cm^2^)** | | | | | | | | | |
|  | **6x6** | | **10x10** | | **15x15** | | **20x20** | | **25x25** | |
|  | **Flat** | **Symm** | **Flat** | **Symm** | **Flat** | **Symm** | **Flat** | **Symm** | **Flat** | **Symm** |
| **2.4** | 9.3 | 0.7 | 4.0 | 1.2 | 2.2 | 0.7 | 1.0 | 0.7 | 1.0 | 0.3 |
| **3.3** | 14.5 | 0.3 | 7.9 | 0.8 | 4.0 | 0.9 | 1.9 | 0.8 | 1.3 | 0.6 |
| **4.3** | 16.7 | 0.5 | 9.6 | 0.7 | 4.8 | 1.2 | 2.2 | 1.3 | 1.1 | 1.0 |
|  | | | | | | | | | | |
|  | | | | | | | | | | |
| **11 MeV Crossline: Flatness and Symmetry (%) Linac 2** | | | | | | | | | | |
| **depth (cm)** | **Cone Size (cm^2^)** | | | | | | | | | |
|  | **6x6** | | **10x10** | | **15x15** | | **20x20** | | **25x25** | |
|  | **Flat** | **Symm** | **Flat** | **Symm** | **Flat** | **Symm** | **Flat** | **Symm** | **Flat** | **Symm** |
| **2.4** | 9.4 | 0.6 | 3.9 | 0.9 | 1.4 | 0.2 | 0.7 | 0.3 | 0.8 | 0.5 |
| **3.3** | 14.6 | 0.5 | 7.8 | 0.8 | 3.3 | 0.3 | 1.4 | 0.3 | 1.2 | 0.6 |
| **4.3** | 16.7 | 1.0 | 9.7 | 0.9 | 4.3 | 0.7 | 1.7 | 0.7 | 1.0 | 0.7 |
